# Birth weight, incident dementia risk, and PET amyloid burden: The ARIC study

**DOI:** 10.1002/alz.70609

**Published:** 2025-09-02

**Authors:** Olivia M. Emanuel, Mark Lee, Pamela L. Lutsey, Kevin J. Sullivan, Renée C. Groechel, Marco Egle, Thomas H. Mosley, Andrea L. C. Schneider, Dean F. Wong, Rebecca F. Gottesman

**Affiliations:** ^1^ National Institute for Neurological Disorders and Stroke National Institutes of Health Bethesda Maryland USA; ^2^ Department of Clinical and Health Psychology University of Florida Gainesville Florida USA; ^3^ Minnesota Department of Health Community Health Division St. Paul Minnesota USA; ^4^ Division of Epidemiology & Community Health University of Minnesota School of Public Health Minneapolis Minnesota USA; ^5^ The MIND Center University of Mississippi Medical Center Jackson Mississippi USA; ^6^ Department of Neurology University of Pennsylvania Perelman School of Medicine Philadelphia Pennsylvania USA; ^7^ Department of Biostatistics, Epidemiology, and Informatics University of Pennsylvania Perelman School of Medicine Philadelphia Pennsylvania USA; ^8^ Department of Radiology Washington University St. Louis Missouri USA

**Keywords:** amyloid, birth weight, dementia, epidemiology, premature birth

## Abstract

**INTRODUCTION:**

The relationships among birth weight (BW), incident dementia, and amyloid beta (Aβ) are unknown.

**METHODS:**

Ten thousand four hundred seventy‐six Atherosclerosis Risk in Communities (ARIC) study participants without dementia reported their BW (1996–1998; low [< 5.5 lbs], medium [5.5–9.0 lbs], or high [> 9.0 lbs]), if premature (yes/no), and were followed for incident dementia (*N* dementia = 2550) through 2019. A subset (*N* = 312) had florbetapir positron emission tomography (2011–2014). BW was evaluated in association with dementia (Cox proportional hazards) and Aβ (logistic regression), adjusted for demographics, apolipoprotein E ε4, and vascular risk. Effect modification by race was explored.

**RESULTS:**

Neither low BW nor prematurity were associated with dementia. Dementia risk was elevated in Black (adjusted hazard ratio 1.95, 95% confidence interval 1.33, 2.86) but not White participants reporting high (versus medium) BW (*P‐*interaction = 0.001). BW was not associated with Aβ.

**DISCUSSION:**

Low BW was not associated with dementia or Aβ. High BW was associated with dementia in Black participants only. Further research should evaluate the role of BW in dementia etiology.

**Highlights:**

- Low birth weight was not associated with an increased risk of incident dementia.
- The association between birth weight and incident dementia was modified by race.
- High birth weight was associated with dementia in Black adults.
- Odds of elevated standardized uptake value ratio was lower for those who were low birth weight.

## 1 BACKGROUND

The global number of dementia cases is projected to rise from 57.4 million in 2019 to 152.8 million by 2050,^1^ highlighting the urgent need to identify risk factors for disease delay and prevention. Early life factors, such as birth weight (BW) and preterm birth, may be an avenue toward this identification. Furthermore, evaluation of BW and related early life factors may be especially important to explain existing disparities in dementia outcomes in individuals from minoritized populations or who were born during periods of particular economic hardship.

BW is frequently used as a proxy for impaired fetal growth, and low BW is related to an increased risk of cardiovascular disease,^2^ hypertension,^3^ and type 2 diabetes mellitus^4^, ^5^ in mid‐ and late life. Preterm birth, also an indicator of impaired fetal growth, has been associated with similar vascular risk factors.^6^, ^7^, ^8^ These risk factors have been connected to mild cognitive impairment and incident dementia in later life;^9^, ^10^ therefore, early life factors such as BW and prematurity may be related to later life cognitive disorders. The impact of early life factors on cognition is multifaceted, necessitating an examination of their role in incident dementia in the context of the cardiovascular pathway.

Furthermore, BW and its sequelae may differentially impact long‐term cognitive health in different groups of individuals, as a reflection of variability in the underlying stressors that individuals from different demographic groups or born during different periods of time experience. Prior research has consistently demonstrated racial disparities in BW, with Black and other minoritized group infants experiencing higher rates of low BW compared to their non‐Hispanic White counterparts.^11^, ^12^, ^13^, ^14^ Thus, exploring differential associations between BW and dementia by race could elucidate some of the underlying reasons for racial differences in dementia rates. Furthermore, socioeconomic and environmental factors, including neighborhood food environments characterized by limited access to nutritious food options and lower socioeconomic status characteristics (e.g., higher poverty rate), have been linked to an increased risk of low BW.^15^, ^16^, ^17^ This might not only impact racial differences in BW and BW‐associated outcomes, but additionally, birth year may also play a critical role in shaping associations between BW and later‐life outcomes, particularly for individuals born during periods of economic hardship, such as the Great Depression in the United States.

Additionally, because early life exposures such as BW and premature birth may influence susceptibility to disease and other risk factors associated with brain pathology in late life,^18^ these exposures may be especially important to consider in the evaluation of brain health because brain pathology is present decades before clinical symptoms of dementia develop.^19^ As part of an evaluation of BW's impact on cognition, it is also important to consider what mechanism might link BW to brain health. Amyloid beta (Aβ) deposition by positron emission tomography (PET) has been established as a biomarker for dementia and is an independent predictor of dementia progression;^20^ thus, understanding how BW relates to Alzheimer's disease (AD) pathology specifically, such as Aβ formation, could explain underlying mechanisms.

For the current study, the Atherosclerosis Risk in Communities (ARIC) Neurocognitive Study (NCS) cohort was used to investigate the association of BW with incident dementia and, in a smaller subset of the cohort, with Aβ burden in late life. We hypothesized that low BW would be associated with subsequent increased risk of incident dementia and Aβ burden in late life. Given other disparities in relation to racial differences in BW,^11^, ^12^, ^13^, ^14^ we hypothesized that these associations may differ by race. We also evaluated differences by birth year, considering that the Great Depression (during, before, and after which many ARIC participants were born) might lead to differences in BW trends and thus the potential importance of low versus medium versus high BW.

## 2 METHODS

### 2.1 Study population

**FIGURE 1 alz70609-fig-0001:**
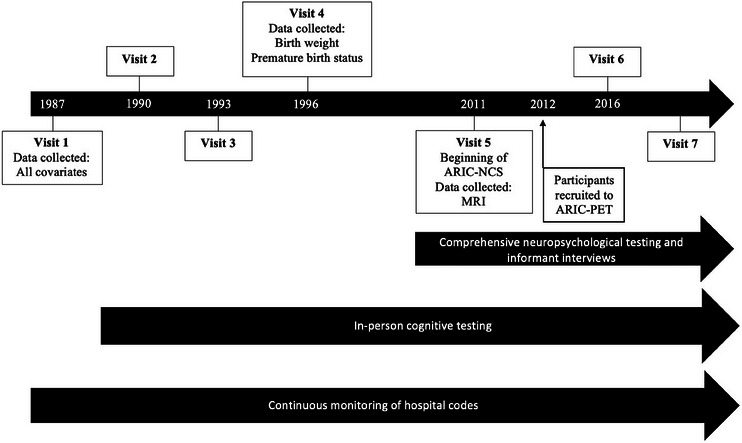
Visual representation of ARIC study design, timeline, and data collection. ARIC, Atherosclerosis Risk in Communities; MRI, magnetic resonance imaging; NCS, Neurocognitive Study; PET, positron emission tomography.

ARIC (Figure 1) is a multi‐center, community‐based prospective cohort study that initially recruited 15,792 participants, aged 45 to 64, between 1987 and 1989.^21^ This cohort sampled from four US communities: Forsyth County, North Carolina; Jackson, Mississippi; northwestern suburban Minneapolis, Minnesota; and Washington County, Maryland. Since baseline, the participants have had continuous surveillance for hospitalizations and death, and numerous follow‐up clinic visits. The first three follow‐up evaluations occurred subsequently at 3 year intervals after the baseline examination (Visits 2, 3, and 4).

At Visit 5 (2011–2013), the ARIC‐NCS began in all surviving participants, and included detailed in‐person neurocognitive evaluation and informant interviews; Visits 6 (2016–2017) and 7 (2018–2019) and 8 (2020; mostly telephone‐based) have followed with a similar comprehensive neuropsychological evaluation. During the entire study duration, there has been surveillance for cardiovascular disease and stroke, and a comprehensive dementia surveillance process was initiated after ARIC Visit 5, including incorporation of data collected from earlier visits, and is ongoing. A subset of participants at ARIC‐NCS Visit 5 had brain magnetic resonance imaging (MRI) scans, who were selected only if the participant had no contraindication to MRI and met one of the following criteria: (1) prior brain MRI during an earlier ARIC brain MRI ancillary visit;^10^ (2) low cognitive scores on a thorough neuropsychological battery from Visit 5 or decline on scores repeated at ARIC Visits 2, 4, and ARIC‐NCS; or (3) an age‐stratified random sample of participants with normal cognition.^22^, ^23^

**RESEARCH IN CONTEXT**

1. **Systematic review**: The authors reviewed the literature using traditional sources (e.g., PubMed) to explore the literature on birth weight (BW) and dementia as well as Alzheimer's disease (AD) neuropathology. Few large representative studies have evaluated these associations and the existing data are inconsistent.
2. **Interpretation**: Our findings suggest that low BW and prematurity are not associated with dementia risk when evaluated in individuals from a US population–based study who survived to late middle age, but that low BW is associated with a lower odds of elevated brain amyloid positron emission tomography, perhaps suggesting a survival bias. Furthermore, BW associations with dementia differed in different race groups and in different birth cohort groups.
3. **Future directions**: Potential mechanisms by which BW might impact dementia aside from through AD‐related pathways should be explored, particularly in those populations in whom low and high BW were found to be associated with dementia risk.

Soon after Visit 5 (within 1 year of the brain MRI scans), a subset of participants without dementia from three centers (Jackson, Mississippi; Washington County, Maryland; Forsyth County, North Carolina) who had brain MRI scans from Visit 5 were recruited into the ARIC‐PET ancillary study and received a florbetapir PET scan (*N* = 346). ARIC‐PET exclusionary criteria have been described in detail.^22^

Participants for the ARIC, ARIC‐NCS, and ARIC‐PET studies provided informed consent at each visit for inclusion in these studies and the studies were approved by the local institutional review boards. The study was performed in accordance with ethical standards as described in the 1964 Declaration of Helsinki and its later amendments. Diversity, equity, and inclusion were prioritized in the design, execution, and interpretation of this study by including participants from diverse racial and geographic backgrounds, addressing health disparities by ensuring equitable access to research.

### 2.2 Early life factor measures

Of the original ARIC cohort, participants seen at ARIC Visit 4 (V4) were included in an ancillary study that incorporated evaluation of self‐reported BW data. Birth information was ascertained by self‐report during a standard interview at ARIC V4 (1996–1998). Participants were asked to report their exact BW in pounds and ounces, but if unable to do so, were asked to select whether their BW was low (< 5.5 lbs), medium (5.5–9.0 lbs), or high (> 9.0 lbs). We excluded 570 total participants who did not provide responses or did not provide sufficient information to categorize their BW (for example, provided number of pounds but not ounces, and a category could not be derived [e.g., 5 lbs, unknown ounces]), and further excluded individuals with prevalent dementia prior to V4 or with missing covariates (Figure S1 in supporting information), leaving an analytic sample for our primary dementia analysis of *N* = 10,746.

Additionally, participants were asked to recall if they were a premature baby, that is, if they were born ≥ 1 month early; an additional 355 participants did not respond or did not know the response to this question, leaving 10,391 participants for this secondary analysis of premature birth status and dementia.[Fig alz70609-fig-0001]

### 2.3 Dementia

Dementia events have been defined in three distinct ways, incorporating data from the entire follow‐up period to estimate events as well as dates of onset.^9^ First, for participants seen in person at Visit 5 or any subsequent ARIC‐NCS visits, dementia diagnoses and dates of onset are defined using expert committee diagnosis from comprehensive in‐person testing (including from visits over the entire study follow‐up) and informant interviews assessing cognitive performance and functional status;^9^, ^24^ 6 months was subtracted from these apparent onset dates when they coincided with study visits to estimate actual date of onset. Additional cases were identified based on telephone‐based cognitive assessment or informant interviews for those participants not seen in person at Visit 5 or later. Finally, hospital codes for dementia were additionally used starting from Visit 1; for individuals with an adjudicated diagnosis based on in‐person evaluation but with an earlier date of onset identified through one of the other two methods, those earlier dates were used. Detailed diagnostic criteria have been previously published.^9^ Dementia cases over the entire duration of follow‐up, through 2020, were used.

### 2.4 Aβ burden

Brain 3T MRI scans were performed at each field center and read centrally at the ARIC MRI Reading Center at the Mayo Clinic; magnetization‐prepared rapid gradient echo images were used to co‐register the PET images. In participants recruited into ARIC‐PET, florbetapir PET scans were performed at three ARIC field centers and images were transferred to the PET image analysis center (Johns Hopkins) for central analysis. Images were quantified for standardized uptake value ratios (SUVRs) in multiple regions of interest, and a global cortical measure of Aβ burden was used for this analysis, representing a weighted average of relevant regions of interest, as previously reported.^22^ As done in prior ARIC studies, global cortical SUVR values were dichotomized at the sample median of SUVR > 1.2,^25^ with “positive” values considered above this threshold. Of the 346 recruited into ARIC‐PET, 310 with complete data including BW self‐report were included for the Aβ deposition analysis (see Figure S2 in supporting information).

### 2.5 Covariate measurements

Covariates include the following for all analyses considering dementia as an outcome: baseline age, sex, a combined variable incorporating both race and field center (race–center: Washington County White; Minneapolis White; Forsyth County White; Forsyth County Black; Jackson Black), education (less than high school; high school/general educational development [GED]/vocational school; or at least some college, graduate, or professional school), birth year cohort (pre‐Great Depression years of birth [up to 1929] vs. during the Great Depression [1929–1939] vs. post‐Great Depression years of birth [after 1939]), cigarette smoking and alcohol consumption status (ever vs. never), continuous body mass index (BMI; kg/m^2^), hypertension (systolic blood pressure > 140 mm Hg, diastolic blood pressure > 90 mm Hg, or taking antihypertensive medications), diabetes (fasting glucose ≥ 126 mg/dL, non‐fasting glucose ≥ 200 mg/dL, HbA1c ≥ 6.5, self‐report of physician‐diagnosed diabetes, or use of oral diabetes medications or insulin), and apolipoprotein E (*APOE*) ε4 status (at least one allele vs. none; TaqMan assay; Applied Biosystems). *APOE* ε4 status was coded with a dummy variable for missing for the dementia incidence analysis, but given the small number with missing genotype in the ARIC‐PET cohort, for that analysis those individuals were excluded (*N* = 3). All variables were considered from the visit at which BWs were ascertained (V4) for both the dementia and the Aβ PET analysis (other than education, race, and *APOE*, which were from Visit 1), and race but not race–center was considered a covariate given small numbers within race–center subgroups in the ARIC‐PET subset.

### 2.6 Statistical analysis

Descriptive analyses compared populations with low, medium, and high BW as well as premature and non‐premature birth for the analytic sample for the dementia analyses as well as for the smaller analytic sample for the ARIC‐PET analysis, using chi‐squared tests and Fisher exact tests for categorical variables, and unadjusted linear regressions for continuous variables.

For the primary dementia analyses, self‐reported BW was assessed, while separate analyses were repeated for self‐reported prematurity (yes/no). Cox proportional hazards models were used to examine the association between self‐reported BW, as well as premature birth status, and time to dementia onset among participants who did not have dementia at ARIC V4; ARIC V4 was considered the start of follow‐up. Follow‐up ended with (1) dementia onset, (2) loss to follow‐up/study withdrawal, or (3) administrative censoring on December 31, 2020. Statistical models were built accordingly, with consideration that many of the potential covariates to be considered might be on the causal pathway between BW and dementia: Model 1 (with no potential mediators) adjusted for age, sex, race–center, and *APOE*; Model 2 added education; and Model 3 added vascular risk factors (hypertension, body mass index, diabetes, cigarette smoking status, and alcohol drinking status).

For the primary Aβ burden analysis, logistic regression models were used to evaluate the association between BW and prematurity, each, and Aβ burden (dichotomous [SUVR > 1.2] florbetapir uptake). Models were built using the same structure as outlined above in the dementia analysis, with the exception of adjustments for race instead of race–center, due to small numbers in ARIC‐PET.

### 2.7 Secondary analyses

Effect modification by race (Black vs. White) and birth cohort (< 1929; 1929–1939; > 1939) were both explored in stratified models and with formal interaction terms for the dementia incidence analysis. In exploratory effect modification analyses, we also evaluated effect modification by hypertension and diabetes by completing stratified analyses with formal testing of interaction terms. Numbers in ARIC‐PET were too small to explore these interactions.

Another secondary analysis evaluated the combined variable incorporating both prematurity status and BW category. The majority of individuals reporting having been premature were in the low BW category, as expected, but we created three categories: first, (the reference category): neither premature nor with low BW; next, having either low BW or having been premature but not both; and finally, having had low BW and having also been born premature.

### 2.8 Sensitivity analysis

The primary analyses (overall, and stratified by race) for BW category and incident dementia were repeated among the much smaller subset of individuals who reported their complete BW in pounds and ounces; they were still categorized into the same three groups but anyone who did not know their exact BW was excluded. In addition, sensitivity analyses were conducted with additional adjustment for income as a measure of socioeconomic status, in the subset with this available, for the overall and year of birth analyses.

As an additional sensitivity analysis, a weighted analysis was conducted in the ARIC‐PET cohort to assess the effect that survivorship may play on the results. Using inverse probability of attrition weighting, participants were weighted back to V4, as this was the time point at which BW data was collected. Weights were created for selection into ARIC‐PET from the V4 sample, with the creation of stabilized weights based on those covariates included in the primary model in addition to other covariates felt to be highly related to selection or survivorship into ARIC‐PET. These weights were then used to repeat the logistic regression analyses for ARIC‐PET.

## 3 RESULTS

### 3.1 Dementia risk analysis study population

Table 1 presents the demographic features and BW distributions of participants included in the dementia incidence analysis. Overall, the population was 54 years old at baseline, 56% were female, and 20% were of Black race. There were 2550 dementia cases identified over a median of 19.5 years (after V4). The majority (92%) of this population had self‐reported medium BW, 6% of this population had self‐reported low BW (6.5% in Black participants vs. 5.6% in White participants), and the remaining 2% (3.2% in Black participants, and 1.7% in White participants) reported high BW. BW differed significantly across race, sex, and education level. Overall, those individuals who provided any BW data were similar to the overall V4 sample, but the subgroup of individuals who gave complete BW data (in pounds and ounces, and not only in a category of BW) were healthier and with higher educational attainment than the total V4 sample or the analytic sample (Table S1 in supporting information).

**TABLE 1:** Demographic features and birth weight distributions from ARIC Visit 4 for participants included in dementia incidence analysis (*N* = 10,746).

Demographic features	Low BW (*n* = 624)	Medium BW (*n* = 9909)	High BW (*n* = 213)	*p* value
Age at V1, y, mean (SD)	53.9 (5.6)	53.9 (5.7)	54.1 (5.4)	0.83
Age at V4, y, mean (SD)	62.8 (5.6)	62.8 (5.7)	63.0 (5.4)	0.85
Race–center, *N* (%)				**<0.001**
*Forsyth County–White (n = 2444)*	135 (21%)	2281 (23%)	32 (15%)	
*Forsyth County–Black (n = 232)*	21 (3%)	207 (2%)	4 (2%)	
*Jackson–Black (n = 1946)*	121 (19%)	1760 (18%)	65 (31%)	
*Washington County–White (n = 2998)*	200 (32%)	2731 (28%)	67 (31%)	
*Minneapolis–White (n = 3126)*	151 (24%)	2930 (30%)	45 (21%)	
Female sex, *N* (%)	438 (70%)	5501 (56%)	63 (30%)	**<0.001**
*APOE* ε4 carrier, *N* (%)				0.86
*Non‐carrier (n = 7222)*	418 (67%)	6661 (67%)	143 (67%)	
*Carrier (n = 3098)*	184 (30%)	2855 (29%)	59 (28%)	
*Missing (n = 426)*	22 (4%)	393 (4%)	11 (5%)	
Education level, *N* (%)				**<0.001**
*< Completed high school (n = 1972)*	146 (23%)	1780 (18%)	46 (22%)	
*High school or equivalent (n = 4557)*	282 (45%)	4193 (42%)	82 (39%)	
*> High school (n = 4217)*	196 (31%)	3936 (40%)	85 (40%)	
Ever smoker, *N* (%)	342 (55%)	5787 (58%)	147 (69%)	**0.001**
Ever alcohol user, *N* (%)	449 (72%)	7930 (80%)	176 (83%)	**<0.001**
BMI at V1, mean (SD) (kg/m^2^)	27.3 (5.5)	27.5 (5.1)	30.0 (5.9)	**<0.001**
BMI at V4, mean (SD) (kg/m^2^)	28.4 (5.9)	28.8 (5.5)	31.2 (6.2)	**<0.001**
Hypertension at V1, *N* (%)	235 (38%)	2974 (30%)	67 (32%)	**<0.001**
Hypertension at V4, *N* (%)	335 (54%)	4617 (47%)	95 (45%)	**0.002**
Diabetes at V1, *N* (%)	58 (9%)	683 (7%)	23 (11%)	**0.009**
Diabetes at V4, *N* (%)	128 (21%)	1589 (16%)	48 (23%)	**0.001**
History of stroke by V4, *N* (%)	15 (2%)	215 (2%)	5 (2%)	0.83
Incident dementia over follow‐up, *N* (%)	166 (27%)	2330 (24%)	54 (25%)	0.18
Premature birth status*, *N* (%)	243 (44%)	59 (< 1%)	1 (< 1%)	**<0.001**

*Note*: Bolded values indicate *p* < 0.05.

Abbreviations: *APOE*, apolipoprotein E; BMI, body mass index; BW, birth weight; SD, standard deviation; V1, Visit 1; V4, Visit 4.

* Premature birth status variable only available on 10,391 participants.

### 3.2 Association of BW with incident dementia

**FIGURE 2:**
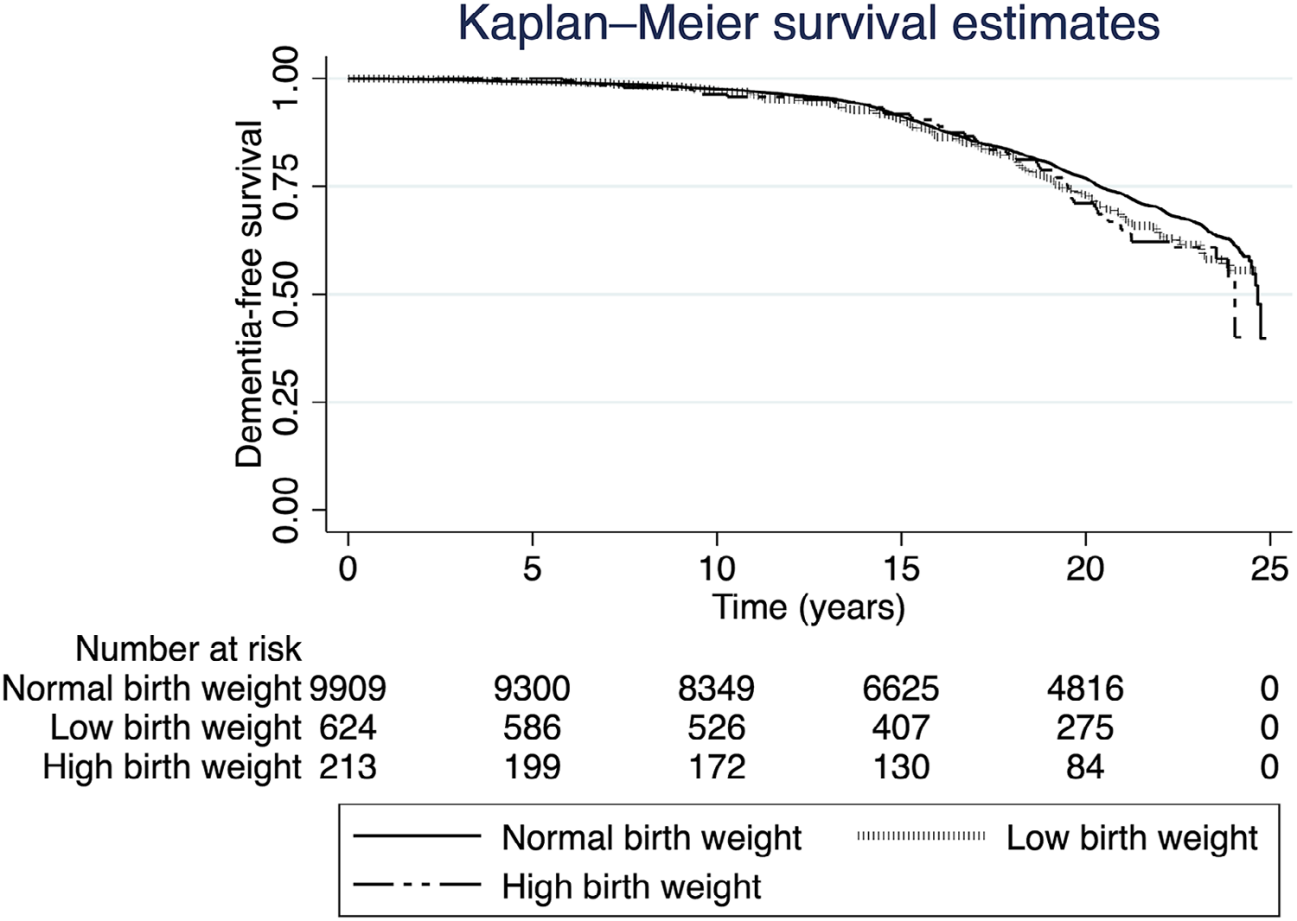
Kaplan–Meier survival curve for the risk of dementia by birth weight category in the Atherosclerosis Risk in Communities study through 2020.

There was no statistically significant difference in dementia‐free survival by BW group in ARIC through 2020 (Figure 2). However, participants who reported a low BW had a non‐significantly (demographic‐adjusted hazard ratio [HR] 1.15, 95% confidence interval [CI] 0.98, 1.35) increased risk of incident dementia compared to those with medium BW (Table 2). This association remained similar, without any statistically significant association, when the statistical model additionally adjusted for education and/or vascular risk factors, which may be mediators of the association (models 2 or 3). Risk of dementia among participants who reported having been born premature (versus not premature) was not significantly elevated.

**TABLE 2:** Hazard ratios of risk of dementia by birth weight in ARIC through 2020.

Independent variables	N events / person‐years	Model 1, HR (95% CI)	Model 2, HR (95% CI)	Model 3, HR (95% CI)
*Dementia by birth weight category*				
Low (< 5.5 lbs.)	166/10574	1.15 (0.98, 1.35)	1.14 (0.98, 1.33)	1.11 (0.95, 1.30)
Medium (5.5–9.0 lbs.)	2330/171619	REF	REF	REF
High (> 9.0 lbs.)	54/3500	1.15 (0.87, 1.50)	1.14 (0.87, 1.49)	1.12 (0.85, 1.47)
*Dementia by premature birth status*				
Non‐premature	2377/174969	REF	REF	REF
Premature	67/5154	1.02 (0.80, 1.31)	1.05 (0.82, 1.34)	1.03 (0.81, 1.32)

*Note*. Model 1: Adjusted for age, sex, race–center, *APOE* ε4 alleles; Model 2: Model 1 + education; Model 3: Model 2 + cigarette use (ever vs. never), alcohol use (ever vs. never), body mass index, hypertension, diabetes.

Abbreviations: *APOE*, apolipoprotein E; ARIC, Atherosclerosis Risk in Communities; CI confidence interval; HR, hazard ratio.

### 3.3 Aβ deposition analysis study population

Table 3 presents the demographic features and BW distributions of participants included in the Aβ deposition analysis (*N* = 312). Overall, at the time of the PET scan the population had a mean (and median) age of 76 years, 56% were female, and 40% were Black. The majority (*N* = 270; 87%) of this population had self‐reported medium BW, with 7% reporting high BW and ≈ 5% (*N* = 17) of this population with self‐reported low BW.

**TABLE 3 alz70609-tbl-0003:** Demographic features and vascular risk factors from Visit 4 in birth weight categories for participants included in amyloid deposition analysis (*N* = 312).

Demographic features	Low BW (*n* = 17)	Medium BW (*n* = 272)	High BW (*n* = 23)	*p* value
Age at V4, years, mean (SD)	63.1 (4.9)	60.8 (5.2)	63.1 (4.4)	**0.03**
Age at PET scan, years, mean (SD)	78.2 (5.2)	75.6 (5.3)	77.9 (4.7)	**0.03**
Black race, *N* (%)	2 (12%)	117 (43%)	6 (26%)	**0.01**
Female sex, *N* (%)	10 (59%)	159 (58%)	7 (30%)	**0.03**
Field center, *N* (%)				0.06
*Forsyth (n = 68)*	5 (29%)	56 (21%)	7 (30%)	
*Jackson (n = 122)*	2 (12%)	114 (42%)	6 (26%)	
*Washington (n = 120)*	10 (59%)	102 (37%)	10 (43%)	
*APOE* ε4 carrier, *N* (%)	3 (18%)	88 (32%)	9 (39%)	0.35
Education level, *N* (%)				0.78
*< Completed high school (n = 48)*	1 (6%)	43 (16%)	4 (17%)	
*High school or equivalent (n = 134)*	7 (41%)	117 (43%)	11 (48%)	
*> High school (n = 128)*	9 (53%)	112 (41%)	8 (35%)	
Current smoker at V4, *N* (%)	0 (0%)	26 (10%)	6 (26%)	0.02
Current alcohol use at V4, *N* (%)	6 (35%)	130 (48%)	11 (48%)	0.65
BMI at V4, mean (SD) (kg/m^2^)	28.6 (4.7)	29.0 (5.3)	29.7 (4.2)	0.76
Hypertension at V4, *N* (%)	5 (29%)	121 (44%)	10 (43%)	0.52
Diabetes at V4, *N* (%)	3 (18%)	32 (12%)	4 (17%)	0.49
Prior Stroke by V4, *N* (%)	0 (0%)	5 (2%)	0 (0%)	1.00
Global cortex Aβ SUVR (mean (SD))	1.19 (0.25)	1.30 (0.25)	1.40 (0.36)	**0.04**
Elevated (SUVR > 1.2) PET Aβ, *N* (%)	4 (24%)	146 (54%)	12 (52%)	0.05
Premature birth status^*^, *N* (%)	8 (50%)	1 (< 1%)	1 (4%)	**<0.001**

*Note*: Bolded values indicate *p* < 0.05;

Abbreviations: Aβ, amyloid beta (from florbetapir positron emission tomography); *APOE*, apolipoprotein E; BMI, body mass index; BW, birth weight; PET, positron emission tomography; SD, standard deviation; SUVR, standardized uptake value ratio; V4, Visit 4.

* Premature birth status only available on 303 participants (16 low BW, 264 medium BW, 23 high BW).

Participants who reported a low BW had 68% (demographic‐adjusted odds ratio [OR] 0.32, 95% CI 0.10, 1.05) non‐significantly *lower* odds of elevated global SUVR compared to participants who reported a medium BW (Table 4), with minimal change in further‐adjusted models. There was no difference in the odds of elevated SUVR between participants who reported high BW versus those that reported medium BW (demographic‐adjusted OR 1.03; 95% CI 0.41, 2.59). The odds of elevated SUVR were also nonsignificantly lower (demographic‐adjusted OR 0.20, 95% CI 0.04, 1.04) in participants who reported having been premature versus not premature (Table 4).

**TABLE 4 alz70609-tbl-0004:** Logistic regression of presence of amyloid deposition in ARIC‐PET participants at Visit 5.

Independent variables	N amyloid positive/ N total	Model 1, OR (95% CI)	Model 2, OR (95% CI)	Model 3, OR (95% CI)
*Birth weight category*				
Low (< 5.5 lbs.)	13/17	0.32 (0.10, 1.05)	0.32 (0.10, 1.06)	0.30 (0.09, 1.02)
Medium (5.5–9.0 lbs.)	146/272	REF	REF	REF
High (> 9.0 lbs.)	12/23	1.03 (0.41, 2.59)	1.04 (0.41, 2.63)	1.01 (0.40, 2.52)
*Premature birth status*				
Non‐premature	156/293	REF	REF	REF
Premature	2/10	0.20 (0.04, 1.04)	0.19 (0.03, 1.02)	0.18 (0.03, 1.04)

*Note*: Model 1: Adjusted for age, sex, race, *APOE* ε4 alleles; Model 2: Model 1 + education; Model 3: Model 2 + body mass index, hypertension, diabetes, smoking, alcohol use.

Abbreviations: *APOE*, apolipoprotein E; ARIC, Atherosclerosis Risk in Communities; CI, confidence interval; OR, odds ratio; PET, positron emission tomography.

### 3.4 Secondary analyses

The association between BW and incident dementia was significantly modified by race (Table S2 in supporting information, Figure 3) in all models (model 3, *p*‐interaction = 0.001). When stratified by race, the risk of incident dementia was elevated in Black participants reporting high (vs. medium) BW (HR 2.08, 95% CI 1.43, 3.04), but not in White participants reporting high BW (HR 0.78, 95% CI 0.52, 1.15), and low BW was associated with elevated risk of dementia in White participants (HR 1.21, 95% CI 1.01, 1.46) but not Black participants (HR 1.09, 95% CI 0.80, 1.48), but only in demographic‐adjusted models (although the interaction remains significant in fully adjusted models, and the HR for dementia remains qualitatively higher in White versus Black participants who had low BW). The risk of dementia did not differ by race for participants reporting premature birth status (model 1 *P*‐interaction = 0.89); White participants with prematurity (versus not premature) model 1 HR 1.04, 95% CI 0.80, 1.35; Black participants with prematurity HR 1.00 (95% CI 0.55, 1.82).[Table alz70609-tbl-0003], [Table alz70609-tbl-0004]

**FIGURE 3 alz70609-fig-0003:**
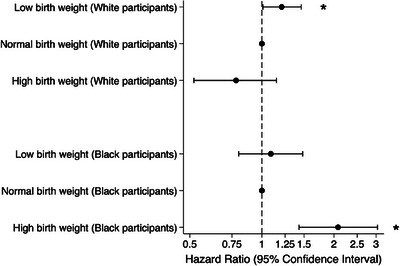
Hazard ratios of incident dementia associated with birth weight categories, stratified by race (*N* = 2177 Black and *N *= 8568 White participants). Model 1: Adjusted for age, sex, apolipoprotein E ε4 alleles; *P*‐interaction = 0.0002. * indicates statistically significant strata (*p* < 0.05).

There was evidence of effect modification of the primary study results by hypertension, but not by diabetes. In individuals with hypertension (Table S3 in supporting information) at the ARIC baseline visit, high BW was associated with dementia in fully adjusted models (model 3; HR 1.86, 95% CI 1.15, 2.99), with no elevated risk associated with low BW. In individuals without hypertension, however, low BW was associated with dementia in models adjusted for demographics and education but not for other vascular risk factors, and high BW had no increased association with dementia. Although associations between both low and high BW and dementia were both qualitatively larger among those with diabetes than among those without diabetes, there was no statistical evidence of significant effect modification by diabetes status (Table S4 in supporting information).[Fig alz70609-fig-0003]

Due to insufficient numbers in race–BW strata in this cohort, stratified analysis of the associations between BW and Aβ deposition by race were unable to be conducted. However, all Black participants who reported low BW (*N* = 2; 100%) had elevated SUVR at Visit 5, compared to only 13% (*N* = 2/15) of White participants who reported low BW (*p* = 0.04, Fisher exact test), and non‐significantly more Black participants with high BW had elevated SUVR (*N* = 4/6, 67%) than did White participants with high BW (*N* = 8/17; 47%; *p* = 0.64).

Analyses were also stratified by birth year (pre‐Great Depression, before 1929 vs. during Great Depression, 1929–1939 vs. post‐Great Depression, after 1939; Table S5 in supporting information), and suggested slightly different associations between BW category and dementia when modified by birth year category (model 3, *P*‐interaction = 0.09). Whereas low BW was associated with dementia among those individuals born during the Great Depression (in model 1 and 2), *high* BW was associated with dementia risk in individuals born after the Great Depression.

When premature birth status and BW category were combined, individuals who were either low BW or premature had an elevated risk of dementia (HR 1.22, 95% CI 1.01, 1.48), with no increase in risk for those with both low BW and premature birth status (Table S6 in supporting information).

### 3.5 Sensitivity analysis

When the primary analyses were repeated among the much smaller sample of individuals who reported complete information on BW in pounds and ounces (*N* = 3744, of whom 344 were categorized as low BW, 82 as high BW, and the remaining 3318 as normal BW), results were similar to the overall analysis (Tables S7 and S8 in supporting information), with similar evidence of interaction by race for the high BW associations, despite the much smaller numbers, and similar effect sizes but attenuation of some of the associations. Sensitivity analyses including additional adjustment for socioeconomic status were similar for the dementia overall and year of birth stratified analyses (Table S9 in supporting information).

The sensitivity analysis using inverse probability of attrition weighting showed similar results to the primary results in the ARIC‐PET sample for the Aβ deposition analysis (see Table S10 in supporting information).

## 4 DISCUSSION

In a community‐based sample, we did not find evidence of an association between self‐reported low BW and incident dementia, but the small number of low BW observations may impact power to detect associations. Low BW was non‐significantly associated with *lower* rates of elevated brain Aβ. Premature birth status was not associated with either dementia or Aβ. Furthermore, BW/dementia associations differed by race: in Black adults, high BW was associated with dementia risk, whereas in White adults, risk was higher for low BW. These results provide insights into the role of BW in the etiology of dementia, and in combination with the Aβ results indicate that associations may reflect non‐AD dementia.

Prior work evaluating BW has been mixed regarding dementia risk. Individuals with lower BW were at higher risk of dementia in the Swedish Twin Registry,^26^ but low BW was not associated with late‐life cognitive outcomes in a Japanese population.^27^ Our study adds to these earlier studies by carefully considering not only potential confounders but also acknowledging that health‐related and lifestyle factors might mediate the hypothesized associations. Interestingly, results only change minimally when these covariates are included, suggesting that any mediation is not substantial.

There are multiple mechanisms potentially linking low BW and dementia. Numerous social factors co‐occur with low BW, which could influence the subsequent development of dementia, such as inadequate access to quality health care or healthy food, and other environmental or social factors. Likewise, BW might contribute to chronic risk factors (e.g., hypertension,^3^ diabetes^5^), which contribute to dementia risk. Interestingly, premature birth was not associated with dementia, but individuals aware of their premature birth status may constitute a distinct subgroup and have better health awareness otherwise. The patterns in the current study are important to validate in more recent cohorts (i.e., participants born after the Great Depression) that include more participants with low BW and with more measurement of social determinants of health.

Associations between BW and health outcomes are inconsistent, often represented by a U‐shaped curve^28^, ^29^, ^30^ whereby both high^4^, ^28^, ^31^, ^32^, ^33^, ^34^, ^35^ and low^32^, ^36^, ^37^, ^38^, ^39^ BW are associated with vascular risk factors in late life. Our finding of high BW being a risk factor in Black but not White adults highlights the need to evaluate these early life factors to pursue mechanisms of underlying disparities in dementia risk. Demographic factors such as race may affect how early life factors influence later life brain health outcomes,^40^, ^41^, ^42^, ^43^ or BW categories may have different meanings by demographic group (e.g., high BW in Black individuals may reflect more pregnancy‐related complications such as gestational diabetes, which itself may indicate risk for worse cognitive performance or other adverse health sequelae^44^). Low BW is more prevalent in Black than White individuals,^11^, ^12^, ^13^, ^14^, ^45^ likely due to systemic racism‐related factors, contributing to disparities in long‐term health outcomes. Thus, the qualitatively increased risk of dementia associated with low BW in White compared to Black participants may reflect a higher risk pregnancy resulting in low BW in White individuals.

BW's association with incident dementia differs depending on birth year relative to the Great Depression. Conditions during those years may have normalized low BW due to limited access to resources, so the finding of low BW being *more* important for individuals born during the Great Depression years, and high BW potentially being more important for dementia risk for those born later, was somewhat unexpected. Those individuals born earlier are obviously older at ARIC visits, and although age is a covariate, these associations may reflect relative differences in basal dementia rates in populations at different risk based on age. Furthermore, the observed differences in BW by year of birth may be influenced by factors unrelated to fetal health, such as differences in BW recall or documentation practices over time.

We found effect modification by hypertension: increased BW was a risk factor for dementia only among individuals with hypertension. These individuals may have worse overall vascular health, or hypertension may have differential severity in individuals with high versus low or normal BW. A similar interaction was not found for diabetes.

In ARIC‐PET, low BW was unexpectedly (non‐significantly) associated with lower odds of elevated SUVR in late life, indicating a protective‐appearing effect on Aβ deposition. Low BW is associated with older brain age on structural MRI^46^ and reduced brain tissue reserve on structural and diffusion tensor MRI,^47^ but studies haven't described associations with Aβ burden. Our unexpected finding may be due to survival bias: ARIC‐PET, by design, excludes people with dementia, potentially biasing away from those with greater risk of poor health. Thus, those individuals who had low BW but were still alive and without dementia may represent a particularly robust subgroup who were resilient to the effects of low BW. Our weighted analysis (weighting the ARIC‐PET sample back to V4), which showed similar results to the primary Aβ analysis, addressed this potential selection bias and attrition.

The existing large studies on birth characteristics and incident dementia are predominantly registry studies^26^ or retrospective surveys^48^ conducted on relatively homogeneous, non‐US populations.^27^ Furthermore, investigations into BW have focused on cardiovascular abnormalities,^2^, ^3^, ^5^, ^37^, ^49^ brain tissue volumes,^47^ and age‐related cognitive impairment.^26^ Our prospective, longitudinal study examines cognitive performance and brain Aβ burden in the context of BW and prematurity status, and evaluates a diverse population. Finally, focusing on all‐cause dementia allows consideration of non‐AD etiologies.

We acknowledge several limitations. The sample size in the ARIC‐PET analysis is relatively small, especially in the low (*n* = 17) and high (*n* = 23) BW groups; therefore, power was especially limited for these analyses. Additionally, bias may be present with the use of self‐report measures; although high validity of self‐reported BW has been established,^50^, ^51^, ^52^ other studies report only fair to moderate agreement between self‐reported and actual BW.^53^ We do not anticipate that bias involving self‐reported BW would be differential with respect to dementia risk, having excluded individuals with prevalent dementia at the time of the BW questionnaire. Our analytic sample was similar to those otherwise evaluated at V4, and our findings remained similar when we considered only individuals giving complete information about their BW; however, the complete BW data group may be subject to selection bias, as they had better health outcomes and higher education attainment. Additionally, the majority of individuals reporting having been born premature (> 80%) were of low BW, strengthening the validity of these self‐reported values. Furthermore, self‐reported BW values in ARIC follow expected biological patterns, with expected sex differences (higher in males than females), and expected correlations with adult‐achieved height and weight.^54^, ^55^ Finally, the rate of low BW in our sample is similar to those reported nationally during a similar time period.^56^

Our community‐based cohort may not be fully representative and the ARIC‐PET substudy, in particular, is subject to selection bias, as discussed above. Furthermore, individuals who developed dementia or died prior to V4 were excluded from both analyses, which may have resulted in fewer individuals with low BW being in our sample, with the remaining group representing a healthier and more robust sample. Both analyses may therefore be susceptible to survivorship bias, and the observed associations between BW and dementia and Aβ burden, each, may underestimate their true relationships. Our analysis is also limited by the absence of data on specific dementia subtypes or on pregnancy or birth‐related complications that might explain differences in BW. Similarly, the absence of tau data limits our understanding to a single biomarker (Aβ) of dementia, precluding a comprehensive evaluation of the relationship between BW and the full spectrum of dementia pathology. Additionally, we acknowledge that there are other social determinants of health that may confound our results. Low BW and high BW, each, may reflect other health‐related changes and are largely impacted by these important social factors (racism, socioeconomic status, risky pregnancy behaviors, marital status,^57^ inadequate prenatal care, and maternal psychological stress are associated with low BW^58^, ^59^, ^60^); our observed differences by race in BW–dementia associations may reflect different social factors in different race groups leading to distinct birth‐related outcomes. Furthermore, low versus high BW may have different meaning and context in the current day, compared to when the participants in this study were born. Finally, we acknowledge that most of the Black participants were from a single study site (Jackson, Mississippi).

We evaluated BW associations with dementia to help focus on early‐life and reproductive health interventions for disease delay and prevention. We did not find evidence that low BW was associated with an increased risk for dementia in the overall population, but selection and survivorship bias could have led to underestimation of true associations. We did, however, find that the associations between BW and risk of incident dementia differed by race, with high BW associated with incident dementia in Black participants only. Last, in a subset of participants, low BW was not associated with elevated Aβ SUVR, a biomarker and a potential link with dementia, on PET imaging, but power was limited. Larger diverse populations should be studied with an emphasis on studies that have directly measured BW, to further evaluate long‐term brain health sequelae of BW.

## CONFLICT OF INTEREST STATEMENT

Author disclosures are available in the supporting information.

## CONSENT STATEMENT

All participants provided informed consent for the described studies.

## Supporting information



Supporting Information

Supporting Information
