# Supplementary material for: Birth weight, incident dementia risk, and PET amyloid burden: The ARIC study
Source: Alzheimers Dement. 2025 Sep 2;21(9):e70609. doi: 10.1002/alz.70609 (PMC12402703; doi:10.1002/alz.70609)
Supplement: Supplementary file 2 — Supporting Information [file ALZ-21-e70609-s001.docx]

**Supplemental Figure 1.** Flowchart of study patient inclusion and exclusion in ARIC-NCS for dementia incidence analysis

**Supplemental Figure 2.** Flowchart of study patient inclusion and exclusion in ARIC-PET for amyloid deposition analysis

**Supplemental Table 1.** Demographic and risk factor for ARIC Visit 4 participants included in dementia incidence analysis, compared to those who provided complete birth weight data, and those seen at visit 4 regardless of whether they included birth weight data

**Supplemental Table 2.** Hazard ratios and 95% confidence intervals of incident dementia associated with birth weight categories stratified by race.

**Supplemental Table 3.** Hazard ratios and 95% confidence intervals of incident dementia associated with birth weight categories stratified by V1 hypertension.

**Supplemental Table 4.** Hazard ratios and 95% confidence intervals of incident dementia associated with birth weight categories stratified by V1 diabetes.

**Supplemental Table 5.** Sensitivity analysis of the modification of birth year (Great Depression: 1929-1939) on associations between birth weight and dementia incidence.

**Supplemental Table 6.** Hazard ratios and 95% confidence intervals of incident dementia associated with combined birth weight and prematurity categories (N=10,391 due to incomplete data for prematurity).

**Supplemental Table 7.** Hazard ratios of risk of dementia by birth weight in ARIC through 2020, among the sample of individuals who reported their complete birth weights (in pounds and ounces).

**Supplemental Table 8.** Hazard ratios and 95% confidence intervals of incident dementia associated with birth weight categories stratified by race, among individuals reporting complete birth weight information (pounds and ounces).

**Supplemental Table 9.** Sensitivity analysis of the modification of birth year (Great Depression: 1929-1939) on associations between birth weight and dementia incidence, additionally adjusted for socioeconomic status.

**Supplemental Table 10.** Logistic regression of presence of amyloid deposition in ARIC-PET weighted population at Visit 5.

**Supplemental Figure 1.** Flowchart of study patient inclusion and exclusion in ARIC-NCS for dementia incidence analysis; V1, Visit 1; WC, Washington County; MN, Minnesota; BW, birth weight; V4, Visit 4; BMI, body mass index.

15,792 participants recruited at V1

**103** excluded: Black participants from WC or MN

**15,689** participants of Black (not from Washington County or Minnesota) or White race

243 patients with GOSE at 30 and/or 90 days

**685** excluded: dementia prior to V4

**15,004** participants without dementia prior to V4

**3427** excluded: not seen at V4 or no follow-up after V4

**11,577** participants were seen at V4 and asked self-reported BW

**144** excluded: missing any BW information

**348** excluded: reported “unknown” BW

**78** excluded: BW reported in 5-lb. range but unknown ounces

**10,883** participants with available APOE data for analysis

**10,746** participants with complete data for dementia incidence analysis

**11,007** participants with self-reported BW at visit 4

**124** excluded: no consent to use DNA

**137** excluded: missing covariates

**Supplemental Figure 2.** Flowchart of study patient inclusion and exclusion in ARIC-PET for amyloid deposition analysis; WC, Washington County; BW, birth weight; V4, Visit 4; V5, Visit 5.

346 ARIC participants recruited to ARIC-PET ancillary study and received PET scans

**1** excluded: had dementia

**345** participants without dementia

**5** excluded: non-Black, non-White, or Black from WC

243 patients with GOSE at 30 and/or 90 days

**340** Black (not from WC) or White participants

**24** excluded: missing BW categorization

**316** participants with self-reported BW (continuous or categorical) at V4

**3** excluded: missing APOE status

**313** participants with available APOE status

**3** excluded: missing diabetes status at V5

**310** participants with available diabetes status at V5

**Supplementary Table 1.** Demographic and risk factor for ARIC Visit 4 participants included in dementia incidence analysis, compared to those who provided complete birth weight data, and those seen at visit 4 regardless of whether they included birth weight data

| **Demographic Features** | **Analytic sample**  **(N = 10,746)** | **Sub-sample who provided BW complete data**  **(N = 3,744)** | **Full visit 4 sample***  **(N = 11,197)** |
| --- | --- | --- | --- |
| Age at V1, y, mean (SD) | 53.9 (5.7) | 53.2 (5.6) | 53.9 (5.7) |
| Age at V4, y, mean (SD) | 62.8 (5.7) | 62.1 (5.6) | 62.8 (5.7) |
| Black Race, N (%) | 2,177 (20.3%) | 595 (15.9%) | 2,414 (21.6%) |
| Race-Center, N (%)  *Forsyth County- White* | 2,444 (22.7%) | 702 (18.8%) | 2,521 (22.5%) |
| *Forsyth County- Black* | 232 (2.2%) | 48 (1.3%) | 249 (2.2%) |
| *Jackson- Black* | 1,946 (18.1%) | 547 (14.6%) | 2,165 (19.3%) |
| *Washington County- White* | 2,998 (27.9%) | 1,281 (34.2%) | 3,083 (27.5%) |
| *Minneapolis- White* | 3,126 (29.1%) | 1,166 (31.1%) | 3,179 (27.5%) |
| Female Sex, N (%) | 6,002 (55.9%) | 2,268 (60.6%) | 6,260 (55.9%) |
| APOE4 Carrier, N (%) |  |  |  |
| *Non-Carrier* | 7,222 (67.2%) | 2,539 (67.8%) | 7,498 (67.0%) |
| *Carrier* | 3,098 (28.8%) | 1,059 (28.3%) | 3,253 (29.1%) |
| *Missing* | 426 (4.0%) | 146 (3.9%) | 446 (4.0%) |
| Education Level, N (%) |  |  |  |
| *< Completed High School* | 1,972 (18.4%) | 564 (15.1%) | 2,142 (19.1%) |
| *High School or equivalent* | 4,557 (42.4%) | 1,588 (42.4%) | 4,725 (42.2%) |
| *> High school* | 4,217 (39.2%) | 1,592 (42.5%) | 4,330 (38.7%) |
| Ever Smoker, N (%) | 6,276 (58.4%) | 2,196 (58.7%) | 6,558 (58.6%) |
| Ever Alcohol User, N (%) | 8,556 (79.6%) | 3,018 (80.6%) | 8,876 (79.3%) |
| BMI at V1, mean (SD) (kg/m^2^) | 27.5 (5.1) | 27.7 (5.4) | 27.5 (5.1) |
| BMI at V4, mean (SD) (kg/m^2^) | 28.8 (5.6) | 29.0 (5.8) | 28.8 (5.6) |
| Hypertension at V1, N(%) | 3,276 (30.6%) | 1,091 (29.3%) | 3,460 (31.0%) |
| Hypertension at V4, N (%) | 5,047 (47.0%) | 1,734 (46.3%) | 5,317 (47.5%) |
| Diabetes at V1, N(%) | 764 (7.1%) | 273 (7.3%) | 819 (7.4%) |
| Diabetes at V4, N (%) | 1,765 (16.4%) | 616 (16.5%) | 1,869 (16.7%) |
| History of Stroke by V4, N (%)** | 235 (2.2%) | 77 (2.1%) | 252 (2.3%) |
| Incident Dementia over followup, N (%) | 2,550 (23.7%) | 830 (22.2%) | 2,686 (24.0%) |
| Birth Weight Category |  |  | N/A |
| *Low* | 624 (5.8%) | 344 (9.2%) |  |
| *Medium* | 9,909 (92.2%) | 3,318 (88.6%) |  |
| *High* | 213 (2.0%) | 82 (2.2%) |  |

*without dementia at visit 4, requiring some followup after v4, with nonmissing key covariates (only excluding those with no BW data)

**missing in 18 individuals, but not covariate in regression models

**Supplemental Table 2.** Hazard ratios and 95% confidence intervals of incident dementia associated with birth weight categories stratified by race (Black, N = 2177; White, N = 8568).

| **Independent Variables** | **N events/ person-years** | **Model 1, HR (95% CI)** | **Model 2, HR (95% CI)** | **Model 3, HR (95% CI)** |
| --- | --- | --- | --- | --- |
| *Dementia by Birth Weight Category (Black participants)* |  |  |  |  |
| Low (<5.5 lbs.) (*n=142*) | 45/2195 | 1.09 (0.80, 1.48) | 1.06 (0.78, 1.43) | 1.05 (0.77, 1.42) |
| Medium (5.5-9.0 lbs.) (*n=1967)* | 557/33082 | REF | REF | REF |
| High (>9.0 lbs.) (*n=69*) | 29/1098 | **2.08 (1.43, 3.04)** | **1.99 (1.36, 2.90)** | **1.95 (1.33, 2.86)** |
| *Dementia by Birth Weight Category (White participants)* |  |  |  |  |
| Low (<5.5 lbs.) (*n=482*) | 121/8379 | **1.21 (1.01, 1.46)** | 1.19 (0.99, 1.43) | 1.16 (0.96, 1.39) |
| Medium (5.5-9.0 lbs.) (*n=7942*) | 177/138538 | REF | REF | REF |
| High (>9.0 lbs.) (*n=144*) | 25/2401 | 0.78 (0.52, 1.15) | 0.76 (0.51, 1.13) | 0.75 (0.51, 1.12) |
| p-interaction |  | **0.0003** | **0.0004** | **0.0010** |

NOTE. Model 1: Adjusted for age, sex, race-center, APOE e4 alleles; Model 2: Model 1 + education; Model 3: Model 2 + cigarette use (ever vs never), alcohol use (ever vs never), body mass index, hypertension, diabetes; Bolded values indicate p<.05

**Supplemental Table 3.** Hazard ratios and 95% confidence intervals of incident dementia associated with birth weight categories stratified by V1 hypertension. (V1 hypertension yes=2,757, no=7,935; total N=10,692 as some missing V1 hypertension information)

| **Independent Variables** | **N events/ person-years** | **Model 1, HR (95% CI)** | **Model 2, HR (95% CI)** | **Model 3, HR (95% CI)** |
| --- | --- | --- | --- | --- |
| *Dementia by Birth Weight Category (No V1 hypertension)* |  |  |  |  |
| Low (<5.5 lbs.) (*n=420*) | 105/7286 | **1.26 (1.03, 1.53)** | **1.24 (1.02, 1.51)** | 1.21 (0.99, 1.48) |
| Medium (5.5-9.0 lbs.) (*n=7363*) | 1635/131676 | REF | REF | REF |
| High (>9.0 lbs.) (*n=152*) | 36/2609 | 1.04 (0.74, 1.44) | 1.01 (0.72, 1.40) | 0.95 (0.68, 1.33) |
| *Dementia by Birth Weight Category (Yes V1 hypertension)* |  |  |  |  |
| Low (<5.5 lbs.) (*n=200*) | 60/3220 | 0.97 (0.74, 1.26) | 0.96 (0.73, 1.25) | 0.95 (0.73, 1.24) |
| Medium (5.5-9.0 lbs.) (*n=2498*) | 684/39047 | REF | REF | REF |
| High (>9.0 lbs.) (*n=59*) | 18/845 | 1.60 (1.00, 2.56) | **1.70 (1.06, 2.72)** | **1.81 (1.13, 2.91)** |
| p-interaction |  | 0.089 | 0.054 | 0.060 |

NOTE. Model 1: Adjusted for V4 age, sex, race-center, APOE e4 alleles; Model 2: Model 1 + education; Model 3: Model 2 + cigarette use (ever vs never), alcohol use (ever vs never), V4 body mass index, V4 diabetes; Bolded values indicate p<.05

**Supplemental Table 4.** Hazard ratios and 95% confidence intervals of incident dementia associated with birth weight categories stratified by V1 diabetes. (V1 diabetes yes=763, no=9,917; total N=10,680 as some missing V1 diabetes information)

| **Independent Variables** | **N events/ person-years** | **Model 1, HR (95% CI)** | **Model 2, HR (95% CI)** | **Model 3, HR (95% CI)** | |
| --- | --- | --- | --- | --- | --- |
| *Dementia by Birth Weight Category (No V1* diabetes*)* |  |  |  |  | |
| Low (<5.5 lbs.) (*n=561*) | 142/9620 | 1.10 (0.93, 1.31) | 1.09 (0.92, 1.29) | 1.07 (0.90, 1.27) | |
| Medium (5.5-9.0 lbs.) (*n=9168*) | 2122/161537 | REF | REF | | REF |
| High (>9.0 lbs.) (*n=188*) | 46/3151 | 1.10 (0.82, 1.47) | 1.10 (0.82, 1.47) | 1.08 (0.80, 1.45) | |
| *Dementia by Birth Weight Category (Yes V1* diabetes*)* |  |  |  |  | |
| Low (<5.5 lbs.) (*n=58*) | 24/881 | 1.50 (0.97, 2.32) | 1.49 (0.96, 2.30) | 1.49 (0.96, 2.31) | |
| Medium (5.5-9.0 lbs.) (*n=683*) | 191/9111 | REF | REF | REF | |
| High (>9.0 lbs.) (*n=23*) | 7/319 | 1.16 (0.53, 2.51) | 1.19 (0.55, 2.57) | 1.21 (0.55, 2.63) | |
| p-interaction |  | 0.449 | 0.383 | 0.407 | |

NOTE. Model 1: Adjusted for V4 age, sex, race-center, APOE e4 alleles; Model 2: Model 1 + education; Model 3: Model 2 + cigarette use (ever vs never), alcohol use (ever vs never), V4 body mass index, V4 hypertension; Bolded values indicate p<.05

**Supplemental Table 5.** Sensitivity analysis of the modification of birth year (Great Depression: 1929-1939) on associations between birth weight and dementia incidence.

| **Independent Variables** | **N events/ person-years** | **Model 1, HR**  **(95% CI)** | **Model 2, HR**  **(95% CI)** | **Model 3, HR**  **(95% CI)** |
| --- | --- | --- | --- | --- |
| *Birth Weight Category by Year of Birth (pre-Great Depression, before 1929); N=2477* |  |  |  |  |
| Low (<5.5 lbs.) (*n=138*) | 54/1896 | 1.02 (0.78, 1.35) | 1.02 (0.77, 1.34) | 0.99 (0.75, 1.31) |
| Medium (5.5-9.0 lbs.) (*n=2291*) | 848/31292 | REF | REF | REF |
| High (>9.0 lbs.) (*n=48*) | 14/630 | 0.95 (0.56, 1.62) | 0.97 (0.57, 1.66) | 0.99 (0.58, 1.69) |
| *Birth Weight Category by Year of Birth (during Great Depression, 1929-1939), N=6084* |  |  |  |  |
| Low (<5.5 lbs.) (*n=365*) | 101/6326 | **1.26 (1.03, 1.55)** | **1.23 (1.00, 1.51)** | 1.21 (0.99, 1.49) |
| Medium (5.5-9.0 lbs.) (*n=5589*) | 1266/99719 | REF | REF | REF |
| High (>9.0 lbs.) (*n=130*) | 31/2215 | 1.08 (0.76, 1.55) | 1.03 (0.72, 1.47) | 1.00 (0.69, 1.43) |
| *Birth Weight Category by Year of Birth (post-Great Depression, after 1939), N=2185* |  |  |  |  |
| Low (<5.5 lbs.) (*n=121*) | 11/2352 | 0.94 (0.51, 1.72) | 0.93 (0.51, 1.71) | 0.92 (0.50, 1.70) |
| Medium (5.5-9.0 lbs.) (*n=2029*) | 216/40608 | REF | REF | REF |
| High (>9.0 lbs.) (*n=35*) | 9/655 | **2.21 (1.12, 4.35)** | **2.11 (1.07, 4.15)** | **2.22 (1.12, 4.39)** |
| p-interaction |  | 0.100 | 0.112 | 0.089 |

Model 1: Adjusted for age, sex, race-center, APOE e4 alleles; Model 2: Model 1 + education; Model 3: Model 2 + cigarette use, alcohol use, body mass index, hypertension, diabetes; Bolded values indicate p<.05

**Supplemental Table 6.** Hazard ratios and 95% confidence intervals of incident dementia associated with combined birth weight and prematurity categories (N=10,391 due to incomplete data for prematurity).

| **Independent Variables** | **Model 1,**  **HR (95% CI)** | **Model 2,**  **HR (95% CI)** | **Model 3,**  **HR (95% CI)** |
| --- | --- | --- | --- |
| *Dementia by Birth Weight and Premature Birth Category* |  |  |  |
| Neither Premature nor Low Birth Weight (*n=9776*) | REF | REF | REF |
| Low Birth Weight *or* Premature (*n=372*) | **1.23 (1.02, 1.49)** | **1.21 (1.00, 1.47)** | **1.22 (1.01, 1.48)** |
| Both Premature *and* Low Birth Weight (*n=243*) | 1.02 (0.77, 1.34) | 1.04 (0.79, 1.37) | 1.00 (0.75, 1.32) |
| NOTE. Model 1: Adjusted for age, sex, race-center, APOE e4 alleles; Model 2: Model 1 + education; Model 3: Model 2 + cigarette use, alcohol use, body mass index, hypertension, diabetes; Bolded values indicate p<.05 | | | |

**Supplemental Table 7.** Hazard ratios of risk of dementia by birth weight in ARIC through 2020, among the sample of individuals who reported their complete birth weights (in pounds and ounces) (N=3,744)*.

| **Independent Variables** | **N events / person-years** | **Model 1,**  **HR (95% CI)** | **Model 2,**  **HR (95% CI)** | **Model 3,**  **HR (95% CI)** |
| --- | --- | --- | --- | --- |
| *Dementia by Birth Weight Category* |  |  |  |  |
| Low (<5.5 lbs.) (*n=344*) | 89/6100 | 1.23 (0.98, 1.53) | 1.22 (0.98, 1.52) | 1.18 (0.94, 1.48) |
| Medium (5.5-9.0 lbs.) (*n=3318*) | 719/58714 | REF | REF | REF |
| High (>9.0 lbs.) (*n=82*) | 22/1292 | 1.19 (0.77, 1.83) | 1.16 (0.76, 1.79) | 1.11 (0.72, 1.71) |
| *Dementia by Premature Birth Status* |  |  |  |  |
| Non-premature (*n=3529)* | 782/62407 | REF | REF | REF |
| Premature (*n=171)* | 37/2989 | 0.98 (0.70, 1.36) | 1.00 (0.72, 1.39) | 0.97 (0.70, 1.35) |
| NOTE. Model 1: Adjusted for age, sex, race-center, APOE e4 alleles; Model 2: Model 1 + education; Model 3: Model 2 + cigarette use (ever vs never), alcohol use (ever vs never), body mass index, hypertension, diabetes; Bolded values indicate p<.05  *N=3,700 for premature status analysis | | | | |

**Supplemental Table 8.** Hazard ratios and 95% confidence intervals of incident dementia associated with birth weight categories stratified by race, among individuals reporting complete birth weight information (pounds and ounces).

| **Independent Variables** | **Model 1, HR (95% CI)** | **Model 2, HR (95% CI)** | **Model 3, HR (95% CI)** |
| --- | --- | --- | --- |
| *Dementia by Birth Weight Category (Black participants)* |  |  |  |
| Low (<5.5 lbs.) (*n=44*) | 1.09 (0.60, 1.96) | 1.06 (0.59, 1.92) | 1.00 (0.56, 1.81) |
| Medium (5.5-9.0 lbs.) (*n=520*) | REF | REF | REF |
| High (>9.0 lbs.) (*n=31*) | **2.11 (1.20, 3.69)** | **2.03 (1.16, 3.56)** | **1.82 (1.02, 3.27)** |
| *Dementia by Birth Weight Category (White participants)* |  |  |  |
| Low (<5.5 lbs.) (*n=300*) | 1.25 (0.98, 1.59) | 1.25 (0.98, 1.59) | 1.23 (0.97, 1.57) |
| Medium (5.5-9.0 lbs.) (*n=2798*) | REF | REF | REF |
| High (>9.0 lbs.) (*n=51*) | 0.69 (0.34, 1.40) | 0.67 (0.33, 1.35) | 0.66 (0.33, 1.33) |
| p-interaction | **0.026** | **0.022** | **0.040** |
| NOTE. Model 1: Adjusted for age, sex, race-center, APOE e4 alleles; Model 2: Model 1 + education; Model 3: Model 2 + cigarette use (ever vs never), alcohol use (ever vs never), body mass index, hypertension, diabetes; Bolded values indicate p<.05 | | | |

**Supplemental Table 9.** Sensitivity analysis of the modification of birth year (Great Depression: 1929-1939) on associations between birth weight and dementia incidence, additionally adjusted for socioeconomic status (N=8,232).

| **Independent Variables** | **Total sample, Fully adjusted* HR**  **(95% CI)** |
| --- | --- |
| *Birth Weight Category for Entire Sample* |  |
| Low (<5.5 lbs.) | 1.13 (0.96, 1.33) |
| Medium (5.5-9.0 lbs.) | REF |
| High (>9.0 lbs.) | 1.11 (0.84, 1.47) |
| *Birth Weight Category by Year of Birth (pre-Great Depression, before 1929); N=2371* |  |
| Low (<5.5 lbs.) | 1.01 (0.76, 1.33) |
| Medium (5.5-9.0 lbs.) | REF |
| High (>9.0 lbs.) | 0.93 (0.54, 1.62) |
| *Birth Weight Category by Year of Birth (during Great Depression, 1929-1939), N=5860* |  |
| Low (<5.5 lbs.) | **1.24 (1.01, 1.53)** |
| Medium (5.5-9.0 lbs.) | REF |
| High (>9.0 lbs.) | 1.07 (0.74, 1.54) |
| *Birth Weight Category by Year of Birth (post-Great Depression, after 1939), N=2185* |  |
| Low (<5.5 lbs.) | 0.90 (0.47, 1.71) |
| Medium (5.5-9.0 lbs.) | REF |
| High (>9.0 lbs.) | 2.05 (0.94, 4.46) |
| p-interaction | 0.218 |

*Adjusted for age, sex, race-center, APOE e4 alleles, education, cigarette use, alcohol use, body mass index, hypertension, diabetes, income category (socioeconomic status). Bolded values indicate p<0.05

**Supplemental Table 10.** Logistic regression of presence of amyloid deposition in ARIC-PET weighted population at Visit 5.

| **Independent Variable** | **Model 1,**  **OR (95% CI)** | **Model 2,**  **OR (95% CI)** | **Model 3,**  **OR (95% CI)** |
| --- | --- | --- | --- |
| *Birth Weight Category* |  |  |  |
| Low (<5.5 lbs.) | 0.32 (0.10, 1.02) | 0.34 (0.10, 1.12) | 0.32 (0.09, 1.09) |
| Medium (5.5-9.0 lbs.) | REF | REF | REF |
| High (>9.0 lbs.) | 1.12 (0.43, 2.90) | 1.24 (0.47, 3.26) | 1.16 (0.44, 3.06) |
| NOTE. Model 1: Adjusted for age, sex, race, APOE e4 alleles; Model 2: Model 1 + education; Model 3: Model 2 + body mass index, hypertension, diabetes, current smoking; Bolded values indicate p<.05 | | | |
